# Constipation in patients with schizophrenia admitted to the Arrazi psychiatric hospital in Salé

**DOI:** 10.1192/j.eurpsy.2025.2194

**Published:** 2025-08-26

**Authors:** N. Ait Bensaid, F. Laboudi, A. Ouanass

**Affiliations:** 1 arrazi psychiatric hospital; 2 HAS, SALE; 3 HAS, arrazi psychiatric hospital in sale, rgoiRRabaRrat; 4 arrazi psychiatric hospital in sale, sale, Morocco

## Abstract

**Introduction:**

Constipation is a common side effect of antipsychotic drugs [1] that has not received much attention.

In patients treated with clozapine, constipation may be the most common side effect, affecting one in three patients [2, 3].

Cases of delayed detection of symptoms of constipation or inadequate treatment of constipation have resulted in paralytic ileus, faecal impaction, intestinal obstruction and even death [4,5].

However, other risk factors for constipation are also common in people with schizophrenia, such as low socio-economic status, a sedentary lifestyle, and illnesses and medications associated with constipation.

Translated with DeepL.com (free version)

**Objectives:**

To assess constipation in patients with schizophrenia hospitalised at the Arrazi psychiatric hospital in Salé and to study the factors associated with these symptoms: socio-demographics, lifestyle, psychotropic drugs, other drugs and co-morbidities such as diabetes and obesity.

**Methods:**

This was a descriptive cross-sectional study using a questionnaire including sociodemographic and clinical criteria as well as questions on the lifestyle of hospitalised patients with schizophrenia to assess constipation in these patients and study the factors associated with these symptoms.

Exclusion criteria: intellectual disability.

**Results:**

A total of 167 patients admitted to the psychiatric hospital for management of schizophrenia were collected. Approximately 69% were male. Adherence to antipsychotics was poor in most patients. 53% were on haloperidol, 25% on olanzapine and 12% on clozapine. Diabetes was present in 21% of patients. Constipation was present in 57% of patients. Most participants had a lifestyle without physical exercise and a balanced diet.

**Conclusions:**

Constipation is common in patients with schizophrenia on antipsychotics, with the risk of complications sometimes severe. It is recommended that clinicians assess antipsychotic-induced constipation and manage it effectively.

**Disclosure of Interest:**

None Declared

